# The New York Institute of Stomatology

**Published:** 1901-05

**Authors:** 

**Affiliations:** The New York Institute of Stomatology


### Reports of Society Meetings.

### THE NEW YORK INSTITUTE OF STOMATOLOGY.

A REGULAR meeting of the Institute was held on Tuesday evening, February 5, at the office of Dr. J. Morgan Howe, No. 12 West Forty-sixth Street, New York, the Vice-President, Dr. A. H. Brockway, in the chair.

The minutes of the previous meeting were read and approved.

### COMMUNICATIONS ON THEORY AND PRACTICE.

*Suprarenal Capsules in Hemorrhage.*

*Dr. E. H. Raymond.*—I would like to call the attention of the gentlemen present to the great value of desiccated suprarenal capsules, which I have been using for the past three months as a hæmostatic. I feel that the preparation is so valuable that it is almost indispensable. Suprarenal capsules have been used constitutionally and locally for about six years by physicians and surgeons in many forms of inflammation in various parts of the body, also for hay-fever, hyperæmia, asthma, etc.

The suprarenal comes in the form of a tablet, powder, and aqueous extract. I have not found any agent that can compare with them for arresting hemorrhage, nor have I yet had a case where it has failed. While working in cavities between the teeth or around the cervical margins where the gums interfere by bleeding,—in fact, wherever we wish to arrest hemorrhage to enable us to continue our operations and not defer them to some future time,—this valuable preparation seems to meet all the requirements. A great advantage it has over the mineral styptics is that, being of an organic nature, there is no soreness nor sloughing of the parts after its use. Another advantage is that it possesses no disagreeable odor. I think the powder preferable to the aqueous extract, as the latter loses its potency in a few days. It is prepared from the suprarenal glands of healthy sheep. It does not seem to possess antiseptic or anæsthetic properties. Parke, Davis & Co. will supply samples and furnish clinical reports.

*Dr. S. H. McNaughton.*—Is it applicable in cases where there is excessive and continued hemorrhage, as after extraction?

*Dr. Raymond.*—I will answer the question by stating a case which I had yesterday. I found a loose root adjacent to a cavity which was very near the gum. I touched the gum with cocaine and extracted the root. There was profuse bleeding, which did not stop. Finally, I filled a pellet of cotton with the suprarenal capsule powder and placed it in the socket twice. The bleeding stopped almost instantly.

*Dr. C. O. Kimball.*—I would like to ask Dr. Raymond if the desiccation of the gum is so complete that gold may be used as a filling in its immediate neighborhood?

*Dr. Raymond.*—That has been my experience. I have never had any trouble with a recurrence of bleeding after one or two applications of the powder.

*Dr. J. B. Locherty.*—In regard to arterial hemorrhage, would Dr. Raymond apply it in these cases with pressure?

*Dr. Raymond.*—I have never had occasion to apply it with pressure except in one case, where I had a long, loose root to extract.

There was considerable bleeding and the patient was in a hurry. I placed some on a piece of cotton and applied with a little pressure, and after repeating this the bleeding stopped immediately.

*Dr. Locherty.*—Can Dr. Raymond tell us if this suprarenal capsule powder acts by contracting the blood-vessels or by forming a clot?

*Dr. Raymond.*—A clot is formed by the fibrin in the blood causing coagulation. The suprarenal capsules contract the blood-vessels.

*Dr. Kimball.*—We are very much obliged to Dr. Raymond for bringing this before us. It is a point which will no doubt be of much use to us. In this connection, the committee would like to ask the gentlemen of the Institute to be always on the alert for just such practical communications. Any point in his daily practice which any gentleman may think would be of help to others, the committee would like to have him feel free to present.

### *Cases of Replantation.*

Dr. R. W. Hunter, of Greenfield, Mass., read a short paper entitled "A Few Cases of Replantation."

(For Dr. Hunter's paper, see page 313.

#### DISCUSSION.

*Dr. A. H. Brockway.*—In what manner are these teeth retained in position?

*Dr. Hunter.*—By rubber dam and silk ligatures. A square piece of rubber dam has two holes punched in each end. Through these holes a silk ligature is passed making a sort of sling. The ligatures are then attached to the adjoining teeth. If there are no adjoining teeth some other means has to be devised.

*Dr. S. E. Davenport.*—Aside from the success which has attended Dr. Hunter's efforts, the most noticeable thing to me in the paper is the honesty of the essayist. I have attended many meetings of various dental societies, and I never before heard an essayist own that he had perforated a root. A great many perforated roots have been spoken of, but they had all been perforated by the other fellow. I explain this honesty of Dr. Hunter's when I remember that he is a Massachusetts man. I am glad to hear Dr. Hunter say that this should not be resorted to when anything else can be done. It seems to me that he will do well not to count too much upon the success which he has attained in these few cases. When his hair is grayer or thinner he will have learned that there is a large element of lottery connected with this sort of operation. Regarding the method in which Dr. Hunter keeps these teeth in position, he has already told us that he shortens the root. I have wondered if the rubber dam splint applied as he has described does not have a tendency to shorten the tooth by pressure, so constant although slight, and that by the time the tooth is firm it is shorter than it should be.

*Dr. Hunter.*—I have not found it so. The pressure is very slight. I do shorten the end of the root, for the reason that I find it practically impossible to get the tooth back again in position unless this is done. Generally speaking, I remove the ligatures in about a week. The tooth even then is quite sore and a little loose. This looseness enables it to take the correct articulation, when it ultimately becomes firm.

*Dr. Davenport.*—In cases of chronic alveolar abscess, supposing Dr. Hunter were to resort to replantation, would he remove some of the diseased tissue from the socket first?

*Dr. Hunter.*—I have never done this in any case where I have resorted to replantation. The only treatment which I have given the socket was to wash it out with listerine and warm water. I think in most cases of abscess, if the irritating cause, the trouble at the end of the root, be removed, the abscess will heal.

*Dr. L. C. Leroy.*—In cutting off the end of the root, which Dr. Hunter says he does in all cases, does he allow the dentine to remain exposed, or does he cover it with some filling-material?

*Dr. Hunter.*—I simply cut off the end of the root and fill the canal with gutta-percha, and then polish off the end of the root as best I can with disks, finishing with a cuttle-fish disk.

*Dr. Brockway.*—Has Dr. Hunter, in the treatment of pyorrhœa alveolaris, where the teeth were exceedingly loose, ever resorted to extraction and replantation? I have heard that such practice has been adopted with success in some cases.

*Dr. Hunter.*—I have never performed replantation in a case of pyorrhœa alveolaris.

*Dr. Wheeler.*—I would like to answer Dr. Brockway's question. I recently had a very bad case of pyorrhœa alveolaris. One central incisor was an eighth of an inch longer than the other. There was nothing to do but to extract the tooth. I suggested, as an experiment, that the tooth be replanted. Finding the pulp putrescent, I cut off the end of the root, filled the canal at the end with tin-foil, with a trephine cut the socket deeper, and put the tooth back in position, so that it was a little shorter than the other. I ligated it in position and left it for six weeks. The result was excellent.

*Dr. Raymond.*—Would Dr. Hunter attempt to replant teeth in the mouth of a patient where absorption of the alveolus had begun or was in progress?

*Dr. Hunter.*—If the absorption was only the absorption due to age, and not to disease, I see no reason why it should not be done.

*Dr. J. Morgan Howe.*—Speaking from some experience, I would say that replantation is a useful expedient as a last resort, as has been suggested by Dr. Hunter, especially in cases of pyorrhœa, affecting the teeth so as to elongate them, but where the socket has not wasted away to a great extent, where absorption has taken place only on one side of the socket, the other parts remaining comparatively intact. Such teeth can be extracted, the socket deepened, and the teeth replanted with success. I have done this successfully several times. I have one patient, a physician in this city, a man of fifty, for whom I have during the past five years treated three teeth successively in this manner with success. The last one was done two and a half years ago.

*Dr. Kimball.*—Did you find the same success in replanting teeth where the socket was partially absorbed and the tooth loose, and where there could be no hold upon the tooth for more than half the length of the root?

*Dr. Howe.*—I think results in these cases depend a good deal upon the condition of the patient. I have failed when patients have been anæmic, weakly, or aged,—past sixty. It has not been my good fortune to see reproduction of alveolar process, so that chances of success, I think, depend largely upon the amount of process still remaining.

*Dr. C. D. Cook.*—In this case which you have mentioned are there other teeth which are affected with the same disease?

*Dr. Howe.*—Yes, there are others, but I prefer to leave them where they are as long as they will stay. These are not operations to seek. As I have said, I think it advisable only as a last resort.

*Dr. Gillett.*—I would like to ask the President if he has noticed any recurrence in these teeth which have been replanted?

*The President.*—No, not as yet.

*Dr. Gillett.*—The statement has been made within a year or so that teeth so replanted are not subject to pyorrhœa, and a case has been reported in which a tooth had been replanted to cure chronic abscess and subsequently every other tooth in the mouth except this one had been attacked by pyorrhœa.

*Dr. Howe.*—My experience in this case is that these teeth of the patient are the firmest which he has. It is probably only a question of time when they will get loose again, but in the mean time he is having the use of them.

*Dr. Cook.*—I suppose you have all noticed in cases of pyorrhœa that the teeth which have been devitalized are the ones which have remained firm. For this reason devitalization has been recommended in cases of pyorrhœa. I have seen this illustrated recently in the case of two molars side by side, one of which was devitalized but which was perfectly firm, while the other which was a sound tooth was very loose with pyorrhœa.

*Dr. Gillett.*—A case in which, about seven years ago, I replanted four teeth may be of interest. A child accidentally knocked out the upper right cuspid, lateral, and two centrals. The cuspid root was developed for only half its length. I replanted this tooth with the rest, filling the end of the root with tin-foil. To my surprise this cuspid tooth, with its root only half formed, became as firm as the others, and for a time the operation was successful, but it and the lateral came out in about two years. The two centrals were in position the last I heard. It seems to me that a point of real practical value is the preparation of the operating-room, having everything as sterile as if a major surgical operation were to be performed. I can sterilize my operating-room as thoroughly as would be desirable if an operation for appendicitis were to be performed. In this manner I have secured practical benefits with very much lessened soreness after replantation and a more satisfactory healing of the parts.

### *Relative Effect of a Common Environment upon Enamel.*

Dr. Frederick L. Bogue read a paper entitled "Relative Effect of a Common Environment upon Enamel."

(For Dr. Bogue's paper, see page 293.)

#### DISCUSSION.

*Dr. G. S. Allan.*—The paper of Dr. Bogue's puts me in mind of a conversation between Sammy Weller and his father as stated in "Pickwick Papers." The elder Weller had surprised his dutiful son just as he had finished a valentine to his dear Mary, and Sammy, as in duty bound, read his effusion to his father for advice and comfort. The document ended abruptly. "That's rathyer a sudden pull up, ain't it, Sammy?" inquired Mr. Weller. "Not a bit on it," said Sam. "She'll vish there was more, and that's the great art o' letter writin'." I think all will agree with me that the doctor's paper came to too sudden a finish, and that more would have been welcome. The paper touches upon a subject in which we are all very much interested, especially when taken in connection with Dr. Black's paper and his (almost) demonstration showing that environment is the only thing which is to be taken into consideration in studying caries of the teeth.

My impression when I read Dr. Black's paper was that his conclusions were pretty much the same as the Scotch verdict "not proven,"—that he did not take hold of this subject from all sides. We all know, since Dr. Miller demonstrated it, that the destruction of the teeth is caused primarily by germs, which in growing liberate certain by-products of an acid nature, lactic acid being the most common, and these, acting chemically on the lime-salts of the tooth, gradually destroy it. Of course, if this acid is weakened, or if it is mixed with other substances, its action is interfered with, either diminished or intensified; but to view this subject simply from the acid stand-point, and to say that environment only is to be considered, is not a fair statement of the question. The physical and chemical make-up of the tooth must in the nature of things equally influence the action that takes place, and as these vary in relative value in different cases, they are not constant factors.

There are so many elements which enter into the problem that it is hardly worth while taking up time discussing the question. If you dilute an acid the chemical reaction goes on with a certain degree of rapidity. Strengthen the acid, and instead of increasing the reaction it is retarded simply because there is not water sufficient to dissolve off the forming salts. Heat and various other things enter into the problem.

The chemical aspects of the case prove to us that, in spite of Dr. Black's statements, certain teeth are more affected by the same environment than others. Now, as I understand the results of Dr. Bogue's experiments, they strongly indicate that some of Dr. Black's statements need revision. The calcium phosphate of the enamel is not broken up by lactic acid; the calcium carbonate is readily dissolved by it. The phosphate of lime usually greatly preponderates in the make-up of the enamel. I cannot conceive, however, how the relative proportions of these two substances could be ascertained in reference to its distribution in the enamel as well as in the dentine. Some experiments made by Dr. Wells suggest that the carbonate of lime is the cementing substance which holds the enamel-rods together, which latter are composed mostly of the phosphate of lime. This was shown by the fact that when the enamel was exposed to a solution of acetic or lactic acid, the prisms became separated. As this acid attacks only the carbonate of lime, it would seem to prove that this carbonate of lime is the cement substance of the enamel. I think some of Dr. Williams's experiments uphold this theory. And so this paper of Dr. Bogue's goes a great way, I think, to support the views of so many of us that there is something else besides environment to be considered in seeking a full solution of the philosophy of the decay of the teeth.

*Dr. J. Bond Littig.*—It has occurred to me that the former use of human teeth on artificial appliances may have some bearing on the case. There were marked differences as to the length of time which these would last in different mouths. The ravages of decay were such that in some mouths the teeth would be gone in six months. I was wondering, as the article was being read, whether the fact that these teeth had been devitalized would have made any difference. The fact remains, however, that these teeth were liable to decay sometimes quite rapidly. In other mouths they would seem to last for a considerable length of time.

*Dr. Davenport.*—In the October number of the INTERNATIONAL DENTAL JOURNAL there appeared an article by Dr. James Truman, the editor, which has made a great impression upon my mind. The title of the editorial was "A Season of Renewed Effort," and it was in part a plea for a better preparation of articles to be presented before dental societies. By better preparation Dr. Truman meant articles based on personal investigation. Theorizing, no matter how well it might be done, would not compare with the carefully chronicled results of investigation.

I think Dr. Frederick L. Bogue is deserving of great credit for what he has done in this direction. Incidentally he has placed credit upon the Institute, which he has chosen as the society before which these investigations appear. It is a matter of interest that our two essayists to-night were both graduated in the same class from the University of Pennsylvania, and were under Dr. Truman's personal supervision. It has always seemed a little strange to me that Dr. Black should not have included the enamel in his investigations; strange that he should not have considered, at just as much length, the physical characteristics of the enamel as he did the physical characteristics of the dentine. I have thought the condition comparable to the removal of the armor from the soldier of olden times in order to find out how much prodding that soldier could stand without armor when he was accustomed to fight with it. Therefore I am particularly pleased that Dr. Bogue has chosen this field for his investigations, and, if I may divulge a secret, this paper, which to all of us as well as to Dr. Allan seems all too short, is, I believe, only the opening chapter. Dr. Bogue's experiments certainly prove something. The environment chosen for the second series of experiments was the same, and the difference of the action upon these enamel surfaces was so great that it proves to my satisfaction that there is a positive difference in the resisting power of enamel. Dr. Miller in his wonderful work of the last twenty years has often produced artificial decay. He has noticed that decay artificially produced sometimes progresses with far greater rapidity than it ever could in any condition of the mouth. This proved to Dr. Miller's satisfaction that the human mouth has some deterrent principle which retards decay, and Dr. Black, taking up the story at that point, wonders whether it is a microbe of a peculiar sort which neutralizes the effect of the microbes which form caries, or whether it is a chemical condition of the secretions. Just here the recent paper of Dr. Michaels, which was read before the International Congress at Paris, seems to me to be a key possibly to the whole situation. Dr. Black explains in his wonderful series of articles that the oral secretions have never yet been examined nor subjected to a consistent series of investigations to find out their influence upon the teeth. If the experiments begun by Dr. Michaels can be carried on, as I feel sure he will carry them on if he lives, the story will, in my opinion, be a much easier one for us to understand; and having diagnosed the case, we may be able to discover remedies for these conditions.

*Dr. J. Morgan Howe.*—I have been very much interested in Dr. Bogue's experiments, for he has allowed me to know something of them as they have been progressing during the past two years or more, and I feel that the Institute has reason to be proud of Dr. Bogue as one of its members, because I regard the presentation of new facts obtained by investigation as of more value than pages and tomes of theories and personal opinions. The facts that Dr. Bogue has presented are of great importance because they must modify our views, which may have been greatly influenced by the announcements of the conclusions of Drs. Black and Williams. These gentlemen, original investigators, to whom we are greatly indebted for facts, agree in the judgment that the decay of teeth is practically due alone to environment and not to structure. In other words, that the physical and chemical make-up of a tooth made no difference, or practically no difference, but that all the variations in the inception and decay of teeth were due to the conditions of the oral fluids and the development and activity of bacteria. If these opinions are correct, it seems to be a necessary conclusion that the same environment would produce the same result on teeth of different kinds. Dr. Bogue's experiments, it seems to me, disprove that necessary conclusion. As Dr. Bogue has said, it at least puts the matter in the position of being an open question. It is to be hoped that Dr. Bogue will be able to go on with his experiments, furnish us with more facts, and thus give us a better basis on which to form conclusions. The probability is that environment has had a great deal more to do with the variations in the production of decay than we have ever believed heretofore. It is expected, and I believe we have reason to anticipate before long more definite information from Dr. Michaels, of Paris, with regard to the effect of the oral fluids in hastening or retarding decay. When we arrive at conclusions based upon all the facts which we are able to obtain, we will probably begin to consider these conditions from the view-point of specialists in medicine, and look for remedies which will influence oral fluids.

*Dr. Kimball.*—While I do not feel competent to discuss the paper from a scientific point of view, I cannot fail to express my appreciation of the work which has been done by Dr. Bogue and also to corroborate what Dr. Howe and Dr. Davenport have said as to the quality of the work. It seems to me that it is just such investigations which are to advance our profession in any definite direction. It is a very satisfactory thing to find one of our own men taking grounds against men whose position in the professional world is so well assured as that of Drs. Black and Williams, and apparently with success. There was one question that occurred to me, whether he noticed any relation between the variations in decay which were produced and any of the recognized differentia; in other words, if, for instance, the specific gravity of the enamel corresponded in any way to the decay produced, or whether there was any differentiation in the enamels which seemed to correspond with the effect produced upon them in the same environment, thus giving us a possible clue as to the reason why some enamels decay more rapidly than others. I am glad to hear that Dr. Bogue is going on with his investigations, and hope we shall have the pleasure of hearing from him again. We shall watch for with interest and welcome with pleasure further reports.

*Dr. E. A. Bogue.*—The essayist has been properly careful not to make any pronouncement except where proof exists. The fact that the teeth of males contain a larger proportion of phosphate of lime than do the teeth of females, and, conversely, that the teeth of females contain a larger proportion of carbonate of lime, was announced at least fifteen or twenty years ago. That the acid in phosphate of lime has a stronger affinity for its base than any of the organic acids commonly found in the mouth is a chemical fact. It is also a chemical fact that carbonic acid in combination with lime has a weaker affinity by far than phosphoric, and that carbonate of lime is susceptible of disintegration and the reformation of soluble salts by the acids which may be formed in the mouth through fermentation. Our clinical experiences show that a great deal more work is done for females than for males. It is, therefore, not surprising that, with these facts before us, we should have jumped to the conclusion that the structure of the teeth themselves was a predisposing cause against or in favor of decay as the case might be. It ought to be a source of gratification to us to see this question carefully and scientifically examined, in order that definite conclusions may be reached, rather than to have it left for conclusions to be inferentially drawn from the few known facts.

In view of the subject of the evening we may be permitted to recall one fact not directly under discussion. It is emphasized by Drs. Black and Williams, as well as the essayist, yet, singularly enough, not one of them pronounces this fact in unmistakable terms. This fact is, a clean tooth never decays. Now, whether we investigate artificial decay or environment in general, or Dr. Black's hoped-for immunity to decay, or the felt-like plaques of Dr. Williams under which decay progresses, or the production of glycogen by the liver, which, changing into glucose and uniting with the ptyaline of the saliva, furnishes the lactic acid of Dr. Michaels, they all lead back to the same condition,—uncleanliness. That the saliva in its normal condition acts as a diluent, and thereby tends to preserve from decay teeth that are constantly bathed in it, is clinically shown by the great immunity to decay of the six lower front teeth. Lest city practitioners should not completely realize what this means, they have but to consult their country brethren to be assured that where total neglect of the teeth exists, these six lower front teeth remain in the mouth, comparatively or quite free from decay, after the loss of all the others.

*Dr. Allan.*—There is one remark in the discussion of the subject by Dr. Bogue to which I would like to take a slight exception,—namely, that teeth which are thoroughly clean will not decay. Clinically I know of apparently clean teeth that have decayed, teeth which have been kept so absolutely clean that it could not be said that they were not so. I have attributed these cases to the fact that there is something in the enamel which invites decay that is not apparent on the surface. I only state it as an exception to the rule. I believe in general that clean teeth do not decay; in fact, I have very radical opinions on the subject.

*Dr. Howe.*—I think Dr. Black, in an article that appears in the December number of the *Dental Cosmos,* practically made a similar statement,—that a clean tooth does not decay. He has reasoned that the portions of the teeth that are constantly rubbed by mastication are not the portions of teeth which decay, and it is those parts that do not get enough friction to keep them clean that decay. This fact has been in my mind for some time, and I have wondered that it has not been referred to before by men of science and men of practice. I think the apparent exceptions to the rule are only apparent. When decay occurs on apparently clean surfaces of teeth, upon careful examination such surfaces would be found covered with an adhering scum which harbors bacteria, thus generating the acid which is the inciting cause of decay, just as Dr. Williams has shown. I think we can realize how much environment affects teeth by noticing the difference in the beginning and progress of decay in the same individual at different times. The rapidity with which it occurs in the mouths of patients suffering with neurasthenia must have been noticed by most of us. Children in school, young folk in college who are under pressure of their studies, business men, and women during gestation, or, under pressure of social affairs, becoming nervously exhausted, have dental decay manifested in greatly increased degree. We notice, too, in advancing years the same phenomenon. I think in every case we may attribute it to changes in the fluids of the mouth, which favor the proliferation of bacteria, and in their adherence to the tooth surface. But that these environments are not the only causes for differences in the inception and progress of decay we have demonstrated by Dr. Bogue's experiments.

*Dr. F. L. Bogue.*—I think Dr. Littig misunderstood me. The point I wished to emphasize was that it seemed probable that there were factors other than environment influencing the rapidity of the decalcification of enamel. Dr. Kimball asked if the specific gravity had any relation to the rapidity of decalcification. Up to the present time I have not been able to determine positively that it bears any relation.

Adjourned.

FRED. L. BOGUE, M.D., D.D.S.,
*Editor The New York Institute of Stomatology.*

